# Factors related to early readmissions after acute heart failure: a nested case–control study

**DOI:** 10.1186/s12872-022-03029-2

**Published:** 2023-01-12

**Authors:** Susana Garcia-Gutierrez, Ane Villanueva, Iratxe Lafuente, Ibon Rodriguez, Ainara Lozano-Bahamonde, Nekane Murga, Josefina Orus, Emilia Rosa Camacho, Jose María Quintana, Raul Quiros, the ReIC-REDISSEC working group, Jose Juan Onaindia, Jose Fernández-Ruiz, Angela Cacicedo, Vanessa Escobar, Maximino Redondo, Gloria Cabello, Marisa Baré

**Affiliations:** 1 Research Unit, Galdakao-Usansolo University Hospital, Barrio Labeaga s/n, 48960 Galdakao, Vizcaya Spain; 2 Kronikgune Institute for Health Services Research, Barakaldo, Spain; 3 Red de Investigación en Servicios Sanitarios Y Enfermedades Crónicas (REDISSEC), Galdakao, Spain; 4 Red de Investigación en Cronicidad, Atención Primaria y Promoción de la Salud (RICAPPS), Girona, Spain; 5 Faculty of Health Sciences, Medicine Department, University of Deusto, Bilbo, Spain; 6 Fundación Vasca de Innovación e Investigación Sanitarias, BIOEF, Barakaldo, Spain; 7 Cardiology Department, Hospital Galdakao-Usansolo, Galdakao, Spain; 8 Cardiology Department, Hospital Basurto, Bilbo, Spain; 9 Cardiology Department, Hospital Parc Taulí, Sabadell, Spain; 10 Internal Medicine Department, Hospital Costa del Sol, Marbella, Spain

**Keywords:** Acute heart failure, Readmissions, PROMS, Transitional period

## Abstract

**Aims:**

To describe the main characteristics of patients who were readmitted to hospital within 1 month after an index episode for acute decompensated heart failure (ADHF).

**Methods and results:**

This is a nested case–control study in the ReIC cohort, cases being consecutive patients readmitted after hospitalization for an episode of ADHF and matched controls selected from those who were not readmitted. We collected clinical data and also patient-reported outcome measures, including dyspnea, Minnesota Living with Heart Failure Questionnaire (MLHFQ), Tilburg Frailty Indicator (TFI) and Hospital Anxiety and Depression Scale scores, as well as symptoms during a transition period of 1 month after discharge. We created a multivariable conditional logistic regression model. Despite cases consulted more than controls, there were no statistically significant differences in changes in treatment during this first month. Patients with chronic decompensated heart failure were 2.25 [1.25, 4.05] more likely to be readmitted than de novo patients. Previous diagnosis of arrhythmia and time since diagnosis ≥ 3 years, worsening in dyspnea, and changes in MLWHF and TFI scores were significant in the final model.

**Conclusion:**

We present a model with explanatory variables for readmission in the short term for ADHF. Our study shows that in addition to variables classically related to readmission, there are others related to the presence of residual congestion, quality of life and frailty that are determining factors for readmission for heart failure in the first month after discharge.

*Trial Registration*: ClinicalTrials.gov Identifier: NCT03300791. First registration: 03/10/2017.

**Supplementary Information:**

The online version contains supplementary material available at 10.1186/s12872-022-03029-2.

## Introduction

Acute decompensated heart failure [ADHF) is one of the leading causes of hospital admission in Spain and other developed countries. In spite of improvements in its diagnosis, management and treatment, the incidence of heart failure continues to rise, due in part to the increasing age of the population [1].

The natural course of the disease leads to frequent readmissions in most cases and more frequently in the final stages of the disease [1]. For a range of reasons, from the decrease in patient quality of life after each episode to their economic impact, physicians and other stakeholders are interested in the prevention of these readmissions. In relation to this, multiple predictive factors and models of readmission after an episode of acute heart failure have been proposed [2, 3]. Large volumes of diverse electronic data and new statistical methods have improved the predictive power of the models over the past two decades. More work is needed, however, in calibration, external validation, and deployment of such models for clinical use since their predictive performance remains poor [3].

Several authors have identified patient reported outcome measures (PROMs), in particular, self-reported quality of life, psychiatric condition and functioning, to be predictors of readmission and longer readmissions in patients with heart failure, and overall in elderly populations [4–8]. Nonetheless, such outcomes have not been included as predictors in classical readmission models and their role in prognosis compared to clinical signs and symptoms has yet to be assessed.

In this context, we hypothesized that psychosocial factors and other PROMs are playing an important role in the probability of readmission after an episode of acute heart failure. Our goal was to describe the main characteristics of patients who were readmitted to a hospital 1 month after an index episode for ADHF.

## Methods

### Design

Predictive Models of Readmission in Heart Failure-REIC (Clinical trials.gov NCT03300791) is a prospective cohort study. We recruited patients with heart failure discharged from five participating hospitals following admission for ADHF and followed them up for 1 year. In order to assess the differences between patients who were and were not readmitted in the first month after discharge, we carried out a nested case–control study, cases being consecutive readmitted patients and controls those who were not readmitted. We identified controls from the initial cohort and matched them with cases based on age, gender and ejection fraction.

### Selection criteria

We included patients over 18 years admitted for acute heart failure syndrome, which includes acute (de novo) heart failure and acute decompensated heart failure: heart failure previously diagnosed but with progressive or rapid worsening of associated signs and symptoms requiring urgent intervention (ICD-9-CM codes: 428.x; and some from 402.x), who agreed to participate and signed the informed consent form.

We excluded patients who developed an episode of ADHF during admission having been admitted for another cause; were transferred from other hospitals; had a myocardial infarction or stroke in the 4 weeks prior to admission; had a life expectancy of less than 1 year, due to terminal heart failure or any other cause; or were unable to complete the questionnaires even with external help (research assistant, family member, social worker, etc.) due tosensory impairment, dementia or lack of knowledge of the language.

#### Measures collected at the discharge

1. Socio-demographic data: age, sex; and race.
2. Medical history: *“*de novo*”* or chronic decompensated heart failure; time since diagnosis of heart failure in those already diagnosed; cardiovascular risk factors; etiology of heart failure; previous echocardiographic parameters; number of previous admissions and ED visits for heart failure; baseline functional status (NYHA class); comorbidities; previous treatments and procedures; and previous vaccinations (pneumococcus, influenza).
3. Admission: precipitating factors; symptoms; signs; ancillary test results including electro and echocardiographic data; treatments and procedures; functional status at discharge (NYHA class); decompensated comorbidities during admission and any other cardiac complications: acute myocardial infarction, ventricular fibrillation, cardiogenic shock, ventricular fibrillation, or cardiac arrest; other complications: renal, hepatic, or thromboembolic conditions, stroke, nosocomial infection, adverse effects or drug interactions); and length of stay.
4. Patient reported outcome measures: Dyspnea at discharge was measured on a Likert scale ranging from “absence” to “the worst possible” and also an analogue visual scale ranging from 0 (no dyspnea) to 10 (the worst dyspnea imaginable) as well as NYHA class at discharge [9].

Minnesota Living with Heart Failure questionnaire (MLHFQ): The MLHFQ assesses the perceived impact of heart failure and its treatment on the lives of patients. It consists of 21 items that cover the physical, psychological, and social limitations associated with heart failure. The patient's perception is evaluated on a scale ranging from zero (not at all) to five (very much). The total MLHFQ score is obtained by adding up the scores for all 21 items (range of 0–105); the higher the score, the worse the health-related quality of life. It is also possible to calculate scores for physical and emotional dimensions (based on eight and five items respectively). The MLHFQ has been validated for use in Spain [10].

Barthel Index: As a measure of disability in activities of daily living in the present study, we used the 10-item Barthel Index, which considers the following activities: feeding, bathing, grooming, dressing, bowel and bladder control, toilet use, chair transfer, mobility, and stair climbing. The score ranges from 0 (complete dependence) to 100 (complete independence). Each item can be awarded 0, 5, 10, or 15 points. In this study, we used the Barthel index categorized as follows: severe dependence (0–50), moderate dependence (51–75), and mild dependence/independence (76–100), as has been used in previous research with similar objectives to those of our study [11, 12].

Lawton-Brody instrumental activities of daily living (IADL) scale: We used this IADL scale as a measure of disability in instrumental activities. It includes eight groups of activities for women (use of the telephone, shopping, food preparation, housework, laundry, transport, control of medication and ability to handle finances), and for men, only five, as preparing food, doing housework or washing clothes are excluded. For each group, there are four or five response options reflecting different levels of functioning. The score ranges from 0 to 8 for women and from 0 to 5 for men [13].

Tilburg Frailty Indicator (TFI): The TFI is composed of two parts. The first part (Part A) describes various determinants of frailty based on socio-demographic data and health-related questions. The second part (Part B) contains 15 items which measure three frailty domains: physical (8 items), psychological (4 items) and social (3 items). Total and domain scores are derived from Part B. The response options are yes/no/sometimes for four items, and yes/no for the others. All items are eventually dichotomized and scored with 0 or 1 point. Scores are the sum of the scores on the corresponding items. A total score ≥ 5 points indicates frailty [14, 15].

Zarit Burden Interview (ZBI): The short version of the ZBI consists of 12 items and covers 2 domains, personal and role. Each question is scored on a five-point Likert scale from 0 (never) to 4 (almost always), with higher scores representing a greater sense of caregiver burden [16].

Pre-episode mood as measured by the Hospital Anxiety and Depression Scale (HADS): This is a self-administered questionnaire consisting of two 7-item subscales, one for anxiety and one for depression. The items of the anxiety subscale were selected based on the analysis of the Hamilton Rating Scale for Anxiety, avoiding the inclusion of physical symptoms that could be confused by the patient with symptoms of his or her physical illness. The items of the depression subscale focus on the area of anhedonia [17].

#### Measures assessed at readmission or by post at 1 month after discharge in the case of controls

Dyspnea (Likert and analogue visual scales) as well as a question related to change in the intensity of dyspnea compared to that experienced at the time of discharge which was categorized as worsening dyspnea vs no change or improvement.

Use of socio-healthcare services after discharge: ED visits, visits to their primary care provider, specialist visits.

Symptoms related to readmission for congestive symptoms, altered mental status, pain, appetite and ulcers.

Treatment adherence as measured by the Morisky Medication Adherence Scale.

Quality of Life (MLWHF); disability (Barthel index and IADL scale); frailty (TFI); and caregiver burden (ZBI), assessed as described above, and also self-care and social support, as assessed with the European Heart Failure Self-care Behaviour Scale and Duke-UNC Functional Social Support Questionnaire respectively.

#### Outcomes

Short-term (during next month after discharge) readmissions.

### Statistical analysis

First, descriptive statistics were generated including frequency tables for categorical variables and means and standard deviations for continuous variables. The sociodemographic and heart failure history-related variables and the responses to the questionnaires at different times over the study were compared between cases and controls using Chi-square or Fisher’s exact tests for categorical data and Student’s t or non-parametric Wilcoxon tests for continuous variables.

With the aim of identifying patient reported variables related to early readmission after an ADHF episode, univariable conditional logistic regression models were built. Variables with p < 0.20 in the univariable analysis were considered potential explanatory variables in the multivariable conditional logistic regression model. Only factors with p < 0.05 were retained in the final model.

For the final multivariable model, odds ratios (ORs) and 95% confidence intervals (95% CIs) were calculated. The predictive and explanatory accuracy of the model was assessed by the coefficient of determination R^2^ and the area under the receiver operating characteristic curve (AUC) [18], and adjusted for estimates of the probabilities were obtained using the conditional logistic regression model.

All statistical analyses were performed using SAS for Windows statistical software, version 9.4 (SAS Institute, Inc., Carey, NC) and R© software version 4.0.5.

## Results

**Fig. 1:**
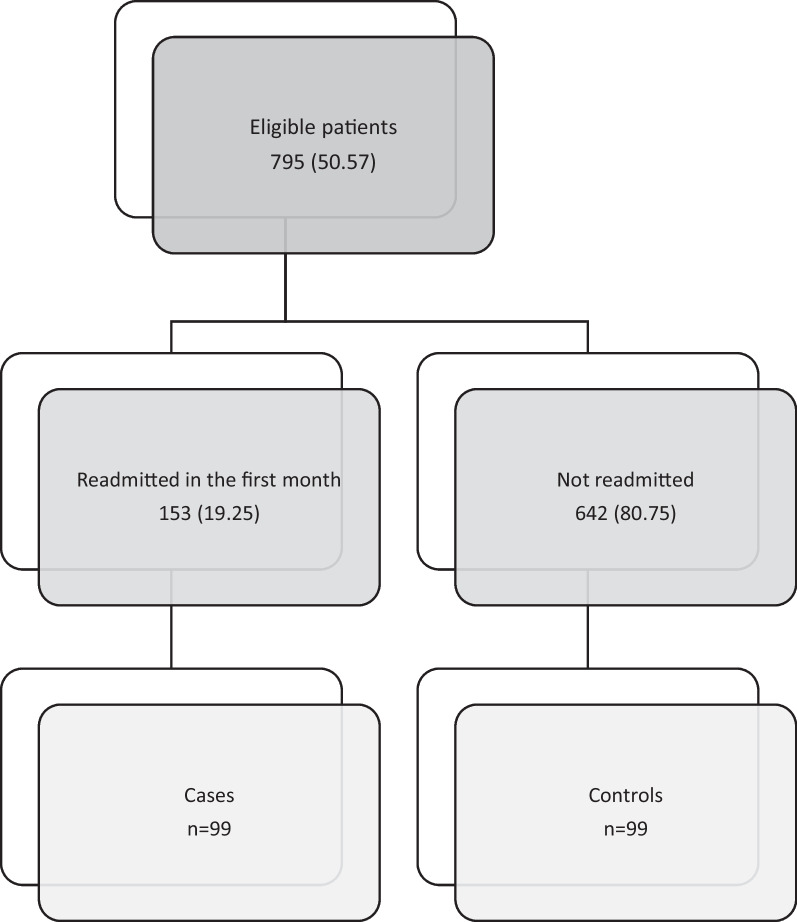
Flow chart

Overall, 795 patients were eligible for the study, and 19% of them were readmitted in the first month after discharge. We recruited 99 cases (64% of readmitted patients) and 99 matched controls from the cohort of non-readmitted patients. Figure 1.

The mean age of our sample was 79.2 (9.2) years and 47% of those recruited were women. More of the cases had chronic decompensation of heart failure (64.5% vs 46.3% of controls, p = 0.01) and had data available on previous echocardiographic parameters (91% vs 79.6% of controls, p = 0.02). Rates of utilization of health services, as reflected in previous readmissions and emergency department visits, were also higher in cases. Lastly, cases were more likely to have undergone an echocardiogram at admission than controls (21% vs 16.3%, p = 0.0003). Table 1.

**Table 1:** Clinical characteristics of cases and controls

Variables	Total	Group		p
	N (%)	Cases N (%)	Controls N (%)	
Total	198	99 (50)	99 (50)	
Sociodemographic variables				
Gender (female)	94 (47.47)	47 (47.47)	47 (47.47)	1.0000
Age*	79.21 (9.2)	79.39 (9.1)	79.03 (9.34)	0.8048
HF history				
Type of acute HF				0.0111
De novo	85 (44.5)	34 (35.42)	51 (53.68)	
Chronic decompensated HF	106 (55.55)	62 (64.58)	44 (46.32)	
Years since diagnosis				0.0163
≥ 3 years	57 (28.93)	36 (36.73)	21 (21.21)	
< 3 years	140 (71.07)	62 (63.27)	78 (78.79)	
Arrhythmias (yes)	116 (59.18)	66 (68.04)	50 (50.51)	0.0125
Information about previous echocardiography (yes)	168 (85.28)	90 (90.91)	78 (79.59)	0.0250
Depressed ejection fraction (< 40%)	46 (23.47)	26 (26.53)	20 (20.41)	0.3119
Ejection fraction				0.5716
< 40	46 (25.00)	26 (28.89)	20 (21.28)	
40–50	28 (15.22)	15 (16.67)	13 (13.83)	
> 50	110 (59.78)	49 (54.44)	61 (64.89)	
Admissions in the 2 previous years for HF (yes)	62 (31.31)	38 (38.38)	24 (24.24)	0.0319
ED visits in the 2 previous years for HF (yes)	25 (12.63)	18 (18.18)	7 (7.07)	0.0186
Charlson comorbidity index*	2.18 (1.83)	2.36 (1.95)	1.98 (1.69)	0.1665
Echo at admission (yes)	115 (58.08)	45 (45.45)	70 (70.71)	0.0003
Dyspnea at discharge (yes)	38 (19.49)	27 (27.55)	11 (11.34)	0.0043
VAS	3.14 (3.95)	3.27 (4.16)	3.02 ( 3.75)	0.65
Likert scale				0.38
Absence	38 (19.39)	16 (16.33)	22 (22.45)	
Mild	90 (45.92)	44 (45)	46 (46.94)	
Moderate	42 (21.43)	20 (20.41)	22 (22.45)	
Severe	21 (10.71)	15 (15.31)	6 (6.12)	
The worst possible	3 (1.53)	2 (2.04)	1 (1.02)	

*HF* heart failure, *EF* ejection fraction, *ED* emergency department, *VAS* visual analogue scale

*Results shown as mean (standard deviation)

Nonetheless, cases and controls did not differ significantly in the following main characteristics: cardiovascular risk factors, vaccination rates, previous cardiac diagnosis, interventions, or comorbidities; nor were there any differences in precipitating factors for decompensation, etiology or functional status at discharge, or education and advice on management of their disease received at discharge. Additional file 1: Table S1 and Additional file 1: Table S2.

**Table 2:** Baseline scores of patient reported outcome measures and change a month after discharge following admission for heart failure

Variables	Baseline*			Change^¥^		
	Cases	Controls	P	Cases	Controls	p
MLHFQ	63.66 (25.26)	59.22 (21.94)	0.1702	3.02 [− 15.34, 13.45]	− 17.37 [− 35,70, − 0.92]	< 0.0001
Physical dimension	28.19 (9.94)	27.75 (9.14)	0.53	0.00 [− 6.00, 6–00]	− 8.00 [− 16.00, 0.00]	< 0.0001
Emotional dimension	15.09 (7.60)	11.96 (6.76)	0.0028	− 1.00 [− 4.00, 3.00]	− 3.00 [− 7.00, 1.00]	0.0814
Tilburg frailty indicator	7.91 (2.79)	7.15 (2.67)	0.0452	0 [− 1.72, 1]	− 0.67 [− 2.29, 1.67]	0.3711
Physical dimension	4.85 (2.08)	4.49 (1.93)	0.1808	− 0.01 (1.76)	− 0.47 (1.98)	0.0758
Psychological dimension	2.11 (1.13)	1.75 (1.17)	0.0301	− 0.04 (1.15)	− 0.11 (1.29)	0.5909
Social dimension	0.93 (0.67)	0.89 (0.78)	0.5354	− 0.10 (0.62)	0.07 (0.68)	0.0579
HADS	19.75 (6.58)	21.26 (6.28)	0.0948	0.3 [− 3.5, 4.17]	2 [− 1, 7]	0.0207
Anxiety scale	10.13 (3.96)	10.87 (3.99)	0.2574	0.00 [− 2.00, 4.00]	1.83 [− 0.10, 4.00]	0.0443
Depression scale	9.54 (3.54)	10.39 (3.14)	0.0663	0.00 [− 1.00, 2.00]	1.00 [− 1.00, 3.00]	0.0974

*MLHFQ* Minnesota Living with Heart Failure Questionnaire, *HADS* Hospital Anxiety and Depression Scale

*Results shown as mean (standard deviation). ^¥^Results shown as median [interquartile range]

Regarding PROMs, cases were more likely to obtain higher scores on the MLHFQ emotional dimension at baseline (15.1 [9.9] vs 11.96 [6.76] in controls, p = 0.0028), though this difference was not clinically meaningful [19]. Similarly, cases scored higher than controls on the TFI psychological dimension (2.11 [1.13] vs 1.75 [1.17] p = 0.03). Table 2.

Nevertheless, the way symptoms changed during the month after discharge differed between cases and controls, cases gaining more weight (probably attributable to heart failure-related fluid retention) and being more likely to present paroxysmal nocturnal dyspnea and congestive symptoms. They were also more likely to report feeling dizzy and having less appetite than controls. Despite cases consulting more with primary care providers than controls, there were no statistically significant differences in changes in treatment during this first month. Table 3. We also analyzed signs and symptoms (including congestive symptoms) at discharge. Just eight patients presented orthopnea or peripheral edema at discharge, three of them being cases and five controls. Nevertheless, changes in congestion during the first month after discharge resulted in more readmissions in the case of those who reported congestive symptoms during this month (p = 0.006). In fact, those who presented congestive symptoms were seen more by their primary care provider but this was not associated with differences in changes in treatment compared to those observed in patients without congestion.Table 4 lists significant variables in univariable analysis. Patients with chronic decompensation of heart failure were more likely (odds ratio: 2.25 (1.25, 4.05)) to be readmitted than de novo patients, as well as to have had admissions or ED visits for heart failure in the previous 2 years. Patients who had not undergone echocardiograms at admission or previously were more likely to be readmitted as well as to have had admissions or ED visits in the 2 previous years. Previous diagnosis of arrhythmia and time since diagnosis ≥ 3 years were significant in the conditional logistic regression model, and patients with worsening in dyspnea, that is, those reporting worse dyspnea than at the time of discharge were 5 times more likely to be readmitted than those who said their condition was the same or better. On the other hand, for every point increase in MLHFQ score, the risk of being admitted increased 4%. Further, for every point increase in TFI score, the risk of being readmitted decreased by 29%.

**Table 3:** Changes in symptoms during the month after discharge

	Total	Group		p
	(%)	Case N (%)	Control N (%)	
Total	198	99 (50)	99 (50)	
Have you had to increase the number of pillows you use for sleeping? (yes)	51 (26.15)	33 (33.33)	18 (18.75)	0.0205
Did you have to sleep sitting up? (yes)	37 (19.27)	26 (26.8)	11 (11.58)	0.0075
Do you wake up at night with fatigue/dyspnea/shortness of breath? (yes)	68 (35.23)	48 (49.48)	20 (20.83)	< 0.0001
Do you often have swelling in your legs? (yes)	105 (53.85)	62 (63.27)	43 (44.33)	0.0080
Have your legs swollen since discharge? (yes)	91 (46.91)	54 (55.1)	37 (38.54)	0.0208
Have your legs swollen more than usual during this month? (yes)	46 (24.21)	34 (35.79)	12 (12.63)	0.0002
Have you noticed any changes in your weight this month?				0.0484
Weight loss	65 (36.93)	35 (42.68)	30 (31.91)	
None	73 (41.48)	26 (31.71)	47 (50)	
Weight gain	38 (21.59)	21 (25.61)	17 (18.09)	
Have you felt dizzy during this time? (yes)	41 (20.92)	27 (27.55)	14 (14.29)	0.0224
Have you had a good appetite since discharge? (yes)	125 (64.77)	55 (56.7)	70 (72.92)	0.0184
Have you consulted your doctor or nurse practitioner about these symptoms? (yes)	67 (39.41)	47 (53.41)	20 (24.39)	0.0001
Has the treatment you were prescribed in hospital been changed? (yes)	50 (26.74)	21 (21.88)	29 (31.87)	0.1228

**Table 4:** Conditional logistic regression model for explanation of short-term readmissions

Variables	Univariable analysis			Multivariable analysis		
	β (SE)	OR (95% CI)	p	β (SE)	OR (95% CI)	p
*Clinical characteristics*						
Chronic decompensation (vs. “de novo” HF)	0.41 (0.15)	2.25 (1.25, 4.05)	0.0070			
Previous admission (2 years) (yes vs. no)	0.29 (0.15)	1.78 (0.99, 3.17)	0.0508			
Previous emergency visits (2 years) (yes vs. no)	0.52 (0.24)	2.83 (1.12, 7.19)	0.0283			
Years of evolution of HF ≥ 3 (vs. < 3)	0.92 (0.37)	2.50 (1.2, 5.21)	0.0143	1.26 (0.58)	3.52 (1.13,11.01)	0.0298
Arrhythmias (yes vs. no)	0.44 (0.17)	2.42 (1.23, 4.74)	0.0101	0.98 (0.49)	2.66 (1.02, 6.93)	0.0444
Echo at admission (yes vs. no)	0.56 (0.17)	3.08 (1.61, 5.91)	0.0007			
Worsening dyspnea (yes vs. no)	1.06(0.41)	2.87(1.29–6.42)	0.0101	1.60 (0.74)	4.96 (1.16, 21.20)	0.0306
*Baseline questionnaires*						
MLHFQ*	0.01 (0.006)	1.01 (0.99, 1.02)	0.1960	0.01 (0.01)	1.01 (0.98,1.04)	0.4614
Tilburg Frailty Indicator*	0.13 (0.06)	1.14 (1.01, 1.29)	0.0328	− 0.06 (0.12)	0.94 (0.73,1.20)	0.6183
HAD*	− 0.03 (0.02)	0.97 (0.93, 1.01)	0.1406	− 0.13 (0.06)	0.88 (0.78,1.00)	0.0519
*Change (1 month score – baseline score)*						
MLHFQ^¥^	0.03 (0.007)	1.03 (1.01, 1.04)	0.0002	0.04 (0.01)	1.04 (1.02, 1.08)	0.0017
Tilburg frailty indicator*	0.04 (0.06)	1.05 (0.94, 1.17)	0.4307	− 0.25 (0.12)	0.77 (0.61, 0.98)	0.0340
HAD*	− 0.05 (0.02)	0.95 (0.91, 0.99)	0.0289	− 0.08 (0.05)	0.92 (0.84, 1.02)	0.1302

β (SE): estimate (standard error). *OR* odds ratio, *CI* confidence interval, *HF* heart failure, *EF* ejection fraction. R2 = 0.23

*Estimation per unit increase

## Discussion

In this study, we have built a model to describe the characteristics of patients who are readmitted in the short term after an episode of ADHF. Time since diagnosis is one of the variables that explains readmissions, those with a more than 3-year history of heart failure being more likely to be readmitted in the short term. Further studies will tell us more about this cut-off point of 3 years in the natural history of the disease. This depiction of the clinical course of heart failure highlights the progressive, nonlinear course of the disease marked by a declining quality of life coupled with increasing care intensity that accelerates after the transition to advanced heart failure [1]. The early identification of patients who are reaching the “advanced” stage could help us to design interventions to mitigate the progression of the disease, as James et al. show to be possible in incident heart failure [20].

Arrhythmias were the only previous cardiac diagnosis found to be related to outcome in the final regression model, in contrast with other authors who have encountered other diagnoses including anemia, peripheral vascular disease, pulmonary hypertension, and valvular heart disease to be possible mechanisms and risk factors for readmission [21]. Arrhythmias are also precipitating factors in heart failure, Wang et al. identifying them as a risk factor for subsequent hospitalizations for heart failure [22]. Arrhythmias, and specifically, atrial fibrillation, are independent risk factors for readmission. It is very important that, before discharge, clinicians establish the most suitable management for each patient given their characteristics, treatment options and tolerance to the arrhythmia itself, with a view to identifying patients who could benefit from a strategy to control the rhythm versus the usual heart rate control strategy.

Readmitted patients were more likely than controls to have congestive symptoms during the month after discharge. In our sample, symptoms of intravascular residual congestion (orthopnea) and tissular congestion (peripheral edema) were more common among patients readmitted but the differences did not reach significance in the multivariable analysis. In contrast, worsening dyspnea was significant in the multivariable analysis. Pang et al. did not encounter a minimal clinically important difference for the 5-point Likert scale we used, but cases were more likely to state that their condition was worse or much worse at the time of readmission [23]. We also analyzed differences in dyspnea as assessed on a visual analogue scale between discharge and the time of readmission or 1 month later for cases (data not shown), but they were neither clinically nor statistically significant. Curiously, in spite of more frequent contact with their primary care provider, their treatment did not change compared to that prescribed at discharge. Given that residual congestion is known to be associated with poor outcomes in heart failure [9, 24], clinicians would be expected to be aware of and treat these symptoms as soon as possible.

Despite no statistically significant differences being observed in PROMS at baseline (discharge), changes in MLHFQ and TFI scores were significantly related to readmission. Cases reported more impact in physical, psychological, and social spheres. Physical and emotional dimensions were both affected, differences being found in items related to tolerance to exercise, e.g., “forced to sit or rest during the day, difficulty walking or climbing stairs, difficulty doing housework/gardening”, as well as those related to social (e.g., “difficulty relating to doing things with family”) and emotional (“feeling worried or depressed”) spheres.

Artalejo et al. showed that poorer quality of life was a predictor of readmission for heart failure in 2005 measured with the SF-36, both physical and mental components being predictors [25]. We have not encountered any more recent studies in which MLHFQ score has been considered a predictor of disease course in heart failure, and hence, lack data with which to compare our results.

Differences in TFI score were also encountered in physical domains as well as in the psychological sphere. Cases were more depressed than controls at baseline, though these differences disappeared 1 month after discharge, and differences were observed in the percentage of patients who presented anxiety during this month. In our sample, improvement in TFI was protective against readmission. Other authors have identified increases in frailty, including TFI score, as a predictor of readmission [26, 27], while some have shown that functioning has better predictive capacity than frailty but we did not find this association in our study [28]]; nor did we find social support to be among the main predictors of readmission [29].

Limitations of the study include those inherent to the case–control design, that is, these findings must be confirmed in further prospective studies. Another limitation was the response rate of the eligible patients, that is, people who responded to the questionnaires needed to be reasonably well to be able to complete them. This has implications for the generalizability of our results. One of the strongest predictor of early readmission in CHF patients is cognitive impairment, formally assesses by a bedside exam like the Mini-Mental or Mini-Cog [30–32]. We aware that there was no possible to measure appropriately cognitive impairment in our real-world study, so Tilburg Failty indicatior single response was considered in the final analysis, being no significant. We encourage authors to include a formal cognitive impairment measures in further studies [33].

In summary, we have developed a model with explanatory variables for readmission in the short term for ADHF. Our study shows that in addition to the variables classically related to readmission (previous admissions, presence of arrhythmias, and duration of heart failure), there are others related to the presence of residual congestion, quality of life and frailty that are determining factors for readmission for heart failure in the first month after discharge.

Further studies are needed to explore whether the discharge transitional period could predict changes in patients’ condition in the medium or long term in the natural course of heart failure.

## Supplementary Information

**Additional file 1. Table 1:** Characteristics of cases and controls. **Table 2.** Descriptive analysis of index episode in cases and controls.

## Data Availability

The datasets used and/or analysed during the current study available from the corresponding author on reasonable request.
