# Supplementary material for: Factors related to early readmissions after acute heart failure: a nested case–control study
Source: BMC Cardiovasc Disord. 2023 Jan 12;23:17. doi: 10.1186/s12872-022-03029-2 (PMC9837935; doi:10.1186/s12872-022-03029-2)
Supplement: Supplementary file 1 — Additional file 1. Table 1: Characteristics of cases and controls. Table 2. Descriptive analysis of index episode in cases and controls. [file 12872_2022_3029_MOESM1_ESM.docx]

Supplementary material:

S1. Table 1: Characteristics of cases and controls:

|  | **Total** | **Group** | | **p-value** | **Missing** |
| --- | --- | --- | --- | --- | --- |
|  | **(%)** | **Cases**  **N (%)** | **Controls**  **N (%)** |  | **(%)** |
| **Total** | **198** | **99 (50)** | **99 (50)** |  |  |
| **Background** |  |  |  |  |  |
| *Demographic characteristics* |  |  |  |  |  |
| Sex (female) | 94 (47.47) | 47 (47.47) | 47 (47.47) | 1.0000 | 0 (0) |
| Age, years* | 79.21 (9.2) | 79.39 (9.1) | 79.03 (9.34) | 0.8048 | 0 (0) |
| Race |  |  |  | Na | 0 (0) |
| Caucasian | 198 (100) | 99 (100) | 99 (100) |  |  |
| Black | 0 (0) | 0 (0) | 0 (0) |  |  |
| Asian | 0 (0) | 0 (0) | 0 (0) |  |  |
| Others | 0 (0) | 0 (0) | 0 (0) |  |  |
| *Index admission* |  |  |  |  |  |
| Acute decompensated heart failure |  |  |  | 0.0111 | 7 (3.54) |
| Chronic decompesated HF | 106 (55.55) | 62 (64.58) | 44 (46.32) |  |  |
| *“De novo”* HF | 85 (44.5) | 34 (35.42) | 51 (53.68) |  |  |
| *Cardiovascular risk factors* |  |  |  |  |  |
| Dyslipidaemia (Yes) | 117 (59.39) | 61 (61.62) | 56 (57.14) | 0.5227 | 1 (0.51) |
| Hypertension (Yes) | 164 (83.25) | 80 (80.81) | 84 (85.71) | 0.3565 | 1 (0.51) |
| Diabetes (Yes) | 89 (45.18) | 44 (44.44) | 45 (45.92) | 0.8354 | 1 (0.51) |
| Hyperuricaemia (Yes) | 36 (18.27) | 20 (20.20) | 16 (16.33) | 0.4816 | 1 (0.51) |
| Hypothyroidism (Yes) | 19 (9.64) | 10 (10.10) | 9 (9.18) | 0.8274 | 1 (0.51) |
| Hyperthyroidism (Yes) | 9 (4.57) | 6 (6.06) | 3 (3.06) | 0.4976 | 1 (0.51) |
| Anaemia (Yes) | 46 (23.35) | 28 (28.28) | 18 (18.37) | 0.1294 | 1 (0.51) |
| Obesity (Yes) | 61 (31.44) | 27 (28.13) | 34 (34.69) | 0.3245 | 4 (2.02) |
| Smoking |  |  |  | 0.2393 | 2 (1.01) |
| Non-smoker | 124 (63.27) | 57 (57.58) | 67 (69.07) |  |  |
| Former smoker | 54 (27.55) | 32 (32.32) | 22 (22.68) |  |  |
| Current smoker | 18 (9.18) | 19 (10.10) | 8 (8.25) |  |  |
| History of alcoholism (Yes) | 23 (11.68) | 9 (9.09) | 14 (14.29) | 0.2563 | 1 (0.51) |
| Other intoxicants (Yes) | 2 (1.02) | 2 (2.04) | 0 (0) | 0.4974 | 2 (1.01) |
| Other cardiovascular risk factors (Yes) | 0 (0) | 0 (0) | 0 (0) | Na | 3 (1.52) |
| Baseline weight, kg | 75.86 (16.92) | 76.85 (18.24) | 74.87 (15.65) | 0.7017 | 112 (56.57) |
| Height, cm* | 161.54 (10.21) | 162.00 (10.46) | 161.10 (10.02) | 0.5698 | 42 (21.21) |
| BMI, kg/m^2^* | 29.59 (4.82) | 29.97 (5.19) | 29.22 (4.45) | 0.7730 | 112 (56.57) |
| *HF history* |  |  |  |  |  |
| Previous echo parameters | 168 (85.28) | 90 (90.91) | 78 (79.59) | 0.0250 | 1 (0.51) |
| EF* | 51.66 (15.59) | 49.57 (17.17) | 54.19 (13.18) | 0.2149 | 79 (39.90) |
| EF |  |  |  | 0.5716 | 79 (39.90) |
| <40 | 26 (21.85) | 16 (24.62) | 10 (18.52) |  |  |
| 40-50 | 22 (18.49) | 13 (20) | 9 (16.67) |  |  |
| >50 | 71 (59.66) | 36 (55.38) | 35 (64.81) |  |  |
| NYHA class at baseline |  |  |  | 0.0093 | 148 (74.75) |
| I | 10 (20) | 5 (14.71) | 5 (31.25) |  |  |
| II | 26 (52) | 15 (44.12) | 11 (68.75) |  |  |
| III | 14 (28) | 14 (41.18) | 0 (0) |  |  |
| IV | 0 (0) | 0 (0) | 0 (0) |  |  |
| Previous admission for HF (2 years) | 62 (31.31) | 38 (38.38) | 24 (24.24) | 0.0319 | 0 (0) |
| Previous emergency department visits for HF (2 years) | 25 (12.63) | 18 (18.18) | 7 (7.07) | 0.0186 | 0 (0) |
| Influenza vaccine in 2017/2018 | 50 (45.87) | 23 (42.59) | 27 (49.09) | 0.4960 | 89 (44.95) |
| Pneumococcal vaccine | 19 (17.92) | 6 (11.54) | 13 (24.07) | 0.0925 | 92 (46.46) |
| Previous diagnoses (yes) | 166 (83.84) | 87 (87.88) | 79 (79.8) | 0.1225 | 0 (0) |
| Myocardial disease (Yes) | 79 (40.31) | 42 (43.3) | 37 (37.37) | 0.3978 | 2 (1.01) |
| Coronary heart disease (Yes) | 45 (23.08) | 21 (21.88) | 24 (24.24) | 0.6949 | 3 (1.52) |
| Hypertensive heart disease (Yes) | 46 (23.59) | 27 (28.13) | 19 (19.19) | 0.1419 | 3 (1.52) |
| Cardiomyopathy (Yes) | 29 (14.87) | 18 (18.75) | 11 (11.11) | 0.1339 | 3 (1.52) |
| Valve disease (Yes) | 79 (40.31) | 45 (46.39) | 34 (34.34) | 0.0856 | 2 (1.01) |
| Arrhythmias (Yes) | 116 (59.18) | 66 (68.04) | 50 (50.51) | 0.0125 | 2 (1.01) |
| Conduction disorders (Yes) | 17 (8.67) | 5 (5.15) | 12 (12.12) | 0.0832 | 2 (1.01) |
| Causes of high cardiac output (Yes) | 2 (1.03) | 1 (1.03) | 1 (1.02) | 1.0000 | 3 (1.52) |
| Pericardial diseases (Yes) | 1 (0.51) | 1 (1.03) | 0 (0) | 0.4949 | 2 (1.01) |
| Diseases of the endocardium (Yes) | 1 (0.51) | 1 (1.03) | 0 (0) | 0.4949 | 2 (1.01) |
| Congenital heart disease (Yes) | 0 (0) | 0 (0) | 0 (0) | Na | 2 (1.01) |
| Volume overload (Yes) | 0 (0) | 0 (0) | 0 (0) | Na | 2 (1.01) |
| Other (Yes) | 8 (4.1) | 6 (6.19) | 2 (2.04) | 0.1695 | 3 (1.52) |
| Previous vascular interventions (Yes) | 79 (39.9) | 38 (38.38) | 41 (41.41) | 0.6633 | 0 (0) |
| *Comorbidities* |  |  |  |  |  |
| Angina (Yes) | 9 (4.55) | 8 (8.08) | 1 (1.01) | 0.0349 | 0 (0) |
| Arrhythmia (Yes) | 104 (52.53) | 58 (58.59) | 46 (46.46) | 0.0877 | 0 (0) |
| Valvular disease (Yes) | 46 (23.23) | 26 (26.26) | 20 (20.20) | 0.3126 | 0 (0) |
| Myocardial Infarction (Yes) | 30 (15.15) | 11 (11.11) | 19 (19.19) | 0.1128 | 0 (0) |
| Congestive HF (Yes) | 95 (47.98) | 54 (54.55) | 41 (41.41) | 0.0644 | 0 (0) |
| Cerebrovascular disease (Yes) | 31 (15.66) | 15 (15.15) | 16 (16.16) | 0.8449 | 0 (0) |
| Hypertension (Yes) | 160 (80.81) | 79 (79.80) | 81 (81.82) | 0.7182 | 0 (0) |
| Peripheral vascular disease (Yes) | 28 (14.14) | 19 (19.19) | 9 (9.09) | 0.0414 | 0 (0) |
| Chronic pulmonary disease |  |  |  | 0.2937 | 0 (0) |
| No | 142 (71.72) | 67 (67.68) | 75 (75.76) |  |  |
| Mild | 32 (16.16) | 20 (20.20) | 12 (12.12) |  |  |
| Moderate/severe | 24 (12.12) | 12 (12.12) | 12 (12.12) |  |  |
| Dementia (Yes) | 3 (1.52) | 1 (1.01) | 2 (2.02) | 1.0000 | 0 (0) |
| Hemiplegia/paraplegia (Yes) | 0 (0) | 0 (0) | 0 (0) | Na | 0 (0) |
| Other neurological diseases (Yes) | 2 (1.01) | 2 (2.02) | 0 (0) | 0.4975 | 0 (0) |
| Diabetes |  |  |  | 02752 | 0 (0) |
| No | 118 (59.60) | 59 (59.60) | 59 (59.60) |  |  |
| Yes, without organ damage | 62 (31.31) | 28 (28.28) | 34 (34.34) |  |  |
| Yes, with organ damage | 18 (9.09) | 12 (12.12) | 6 (6.06) |  |  |
| Other endocrine diseases (Yes) | 0 (0) | 0 (0) | 0 (0) | Na | 0 (0) |
| Renal insufficiency |  |  |  | 0.7859 | 0 (0) |
| No | 179 (90.40) | 90 (90.91) | 89 (89.90) |  |  |
| Mild | 12 (6.06) | 5 (5.05) | 7 (7.07) |  |  |
| Moderate/severe | 7 (3.54) | 4 (4.04) | 3 (3.03) |  |  |
| Gastrointestinal bleeding (Yes) | 11 (5.56) | 6 (6.06) | 5 (5.05) | 0.7564 | 0 (0) |
| Inflammatory bowel disease (Yes) | 2 (1.01) | 2 (2.02) | 0 (0) | 0.4975 | 0 (0) |
| Peptic ulcer (Yes) | 12 (6.09) | 4 (4.08) | 8 (8.08) | 0.2406 | 1 (0.51) |
| Tumour (Yes) | 14 (7.07) | 9 (9.09) | 5 (5.05) | 0.2674 | 0 (0) |
| Lymphoma (Yes) | 0 (0) | 0 (0) | 0 (0) | Na | 0 (0) |
| Leukaemia (Yes) | 1 (0.51) | 1 (1.01) | 0 (0) | 1.0000 | 0 (0) |
| AIDS (Yes) | 0 (0) | 0 (0) | 0 (0) | Na | 0 (0) |
| Metastatic cancer (Yes) | 2 (1.01) | 1 (1.01) | 1 (1.01) | 1.0000 | 0 (0) |
| Liver disease |  |  |  | 0.8443 | 0 (0) |
| No | 191 (96.46) | 96 (96.97) | 95 (95.96) |  |  |
| Mild | 3 (1.52) | 1 (1.01) | 2 (2.02) |  |  |
| Moderate/Severe | 4 (2.02) | 2 (2.02) | 2 (2.02) |  |  |
| Rheumatological disease (Yes) | 9 (4.55) | 7 (7.07) | 2 (2.02) | 0.1696 | 0 (0) |
| Coagulopathy (Yes) | 68 (34.34) | 34 (34.34) | 34 (34.34) | 1.0000 | 0 (0) |
| Hyponatremia (Yes) | 1 (0.51) | 1 (1.01) | 0 (0) | 1.0000 | 0 (0) |
| Anaemia (Yes) | 42 (21.21) | 25 (25.25) | 17 (17.17) | 0.1643 | 0 (0) |
| Iron deficiency (Yes) | 24 (12.18) | 15 (15.15) | 9 (9.18) | 0.2004 | 1 (0.51) |
| Hyperthyroidism (Yes) | 7 (3.54) | 4 (4.04) | 3 (3.03) | 1.0000 | 0 (0) |
| Hypothyroidism (Yes) | 18 (9.09) | 10 (10.10) | 8 (8.08) | 0.6210 | 0 (0) |
| Sleep disorders/obstructive sleep apnoea (Yes) | 5 (2.53) | 2 (2.02) | 3 (3.03) | 1.0000 | 0 (0) |
| Anxiety (Yes) | 19 (9.60) | 10 (10.10) | 9 (9.09) | 0.8093 | 0 (0) |
| Depression (Yes) | 28 (14.14) | 12 812.12) | 16 (16.16) | 0.4146 | 0 (0) |
| Mild cognitive impairment (Yes) | 3 (1.52) | 2 (2.02) | 1 (1.01) | 1.0000 | 0 (0) |
| Psychiatric pathology (Yes) | 3 (1.52) | 2 (2.02) | 1 (1.01) | 1.0000 | 0 (0) |
| Charlson Index* (Yes) | 2.18 (1.83) | 2.36 (1.95) | 1.98 (1.69) | 0.1665 | 0 (0) |

*¥Results shown as mean (standard deviation). ¥Results shown as median [interquartile range]. Na: It is not possible to calculate the p-value. HF: heart failure. BMI: body mass index. EF: ejection fraction. NYHA: New York Heart Association classification system for heart failure.

**S2. Table 2.** Descriptive analysis of index episode in cases and controls

|  | **Total** | **Grupo** | | **p-valor** | **Missing** |
| --- | --- | --- | --- | --- | --- |
|  | **(%)** | **Caso**  **N (%)** | **Control**  **N (%)** |  | **(%)** |
| **Total** | **198** | **99 (50)** | **99 (50)** |  |  |
| Echocardiogram on admission (Yes) | 115 (58.08) | 45 (45.45) | 70 (70.71) | 0.0003 | 0 (0) |
| Admission EF* | 49.53 (18.46) | 45.07 (20.92) | 52.32 (16.28) | 0.1165 | 89 (44.95) |
| *Precipitant factors* |  |  |  |  |  |
| Mechanical causes (Yes) | 0 (0) | 0 (0) | 0 (0) | Na | 0 (0) |
| Stroke (Yes) | 0 (0) | 0 (0) | 0 (0) | Na | 0 (0) |
| Surgery and perioperative complications (Yes) | 0 (0) | 0 (0) | 0 (0) | Na | 0 (0) |
| Therapeutic non-compliance (Yes) | 3 (1.52) | 3 (3.03) | 0 (0) | 0.2462 | 0 (0) |
| Recent change of treatment (Yes) (Yes) | 2 (1.01) | 1 (1.01) | 1 (1.01) | 1.0000 | 0 (0) |
| Use of vasopressors (Yes) | 1 (0.51) | 1 (1.01) | 0 (0) | 1.0000 | 0 (0) |
| Use of NSAIDs (Yes) | 1 (0.51) | 0 (0) | 1 (1.01) | 1.0000 | 0 (0) |
| Use of corticoids (Yes) | 0 (0) | 0 (0) | 0 (0) | Na | 1 (0.51) |
| Increased metabolic demands (Yes) | 15 (7.58) | 9 (9.09) | 6 (6.06) | 0.4204 | 0 (0) |
| Pulmonary embolism (Yes) | 0 (0) | 0 (0) | 0 (0) | Na | 0 (0) |
| Acute myocardial infarction (Yes) | 3 (1.52) | 1 (1.01) | 2 (2.02) | 1.0000 | 0 (0) |
| Uncontrolled hypertension (Yes) | 10 (5.05) | 6 (6.06) | 4 (4.04) | 0.5163 | 0 (0) |
| Infective endocarditis, myocarditis (Yes) | 0 (0) | 0 (0) | 0 (0) | Na | 0 (0) |
| Respiratory infection (Yes) | 43 (21.72) | 24 (24.24) | 19 (19.19) | 0.3888 | 0 (0) |
| Pneumonia (Yes) | 4 (2.02) | 0 (0) | 4 (4.04) | 0.1212 | 0 (0) |
| COPD (Yes) | 4 (2.02) | 3 (3.03) | 1 (1.01) | 0.6212 | 0 (0) |
| Cor pulmonale (Yes) | 0 (0) | 0 (0) | 0 (0) | Na | 0 (0) |
| Intercurrent diseases (Yes) | 16 (8.08) | 5 (5.05) | 11 (11.11) | 0.1177 | 0 (0) |
| Rapid atrial fibrillation (Yes) | 48 (24.24) | 24 (24.24) | 24 (24.24) | 1.0000 | 0 (0) |
| Loss of sinus rhythm (Yes) | 7 (3.54) | 3 (3.03) | 4 (4.04) | 1.0000 | 0 (0) |
| Unknown (Yes) | 59 (29.8) | 28 (28.28) | 31 (31.31) | 0.6411 | 0 (0) |
| Toxic Use (Yes) | 0 (0) | 0 (0) | 0 (0) | Na | 0 (0) |
| Dietary violations (Yes) | 0 (0) | 0 (0) | 0 (0) | Na | 0 (0) |
| Iatrogenic volume overload (Yes) | 0 (0) | 0 (0) | 0 (0) | Na | 0 (0) |
| Other cause (Yes) | 2 (1.01) | 1 (1.01) | 1 (1.01) | 1.0000 | 0 (0) |
| *Signs and symptoms* |  |  |  |  |  |
| Altered level of consciousness/confusion (Yes) | 5 (2.53) | 2 (2.02) | 3 (3.03) | 1.0000 | 0 (0) |
| Cachexia (Yes) | 0 (0) | 0 (0) | 0 (0) | Na | 0 (0) |
| Sarcopenia (Yes) | 0 (0) | 0 (0) | 0 (0) | Na | 0 (0) |
| Orthopnea (Yes) | 70 (35.53) | 38 (38.78) | 32 (32.32) | 0.3441 | 1 (0.51) |
| Paroxysmal nocturnal dyspnoea (Yes) | 25 (12.63) | 11 (11.11) | 14 (14.14) | 0.5209 | 0 (0) |
| Incontinence (Yes) | 10 (5.05) | 5 (5.05) | 5 (5.05) | 1.0000 | 0 (0) |
| Oliguria (Yes) | 52 (26.26) | 27 (27.27) | 25 (25.25) | 0.7467 | 0 (0) |
| Precordial pain (Yes) | 3 (1.52) | 1 (1.01) | 2 (2.02) | 1.0000 | 0 (0) |
| Vegetative Cortex (Yes) | 1 (0.51) | 0 (0) | 1 (1.01) | 1.0000 | 0 (0) |
| Cyanosis (Yes) | 4 (2.02) | 2 (2.02) | 2 (2.02) | 1.0000 | 0 (0) |
| Chills (Yes) | 4 (2.02) | 2 (2.02) | 2 (2.02) | 1.0000 | 0 (0) |
| Pallor (Yes) | 26 (13.13) | 16 (16.16) | 10 (10.1) | 0.2068 | 0 (0) |
| Bilateral oedema with pitting (Yes) | 134 (67.68) | 62 (62.63) | 72 (72.73) | 0.1286 | 0 (0) |
| Hepatomegaly (Yes) | 1 (0.51) | 1 (1.01) | 0 (0) | 1.0000 | 0 (0) |
| Ascites (Yes) | 5 (2.53) | 3 (3.03) | 2 (2.02) | 1.0000 | 0 (0) |
| Jugular ingurgitation (Yes) | 52 (26.53) | 23 (23.71) | 29 (29.29) | 0.3762 | 2 (1.01) |
| Heart rate* (Yes) | 89.79 (26.83) | 90.56 (25.8) | 89.02 (27.95) | 0.4601 | 3 (1.52) |
| Respiratory rate* (Yes) | 21.86 (5.27) | 22.7 (5.98) | 21 (4.31) | 0.1607 | 68 (34.34) |
| Temperature* | 36.46 (0.41) | 36.43 (0.4) | 36.49 (0.41) | 0.1093 | 17 (8.59) |
| O2 saturation* | 92.68 (6.52) | 93.11 (5.26) | 92.27 (7.55) | 0.6105 | 3 (1.52) |
| FiO2* | 23.44 (12.76) | 23.02 (11.36) | 23.8 (13.97) | 0.8395 | 93 (46.97) |
| Weight* | 85.24 (33.58) | 94.98 (39.29) | 69 (15.1) | 0.2967 | 190 (95.96) |
| TAS* | 142.25 (27.64) | 141.08 (28.11) | 143.4 (27.26) | 0.3662 | 1 (0.51) |
| TAD* TAD | 78.95 (17.05) | 79.99 (16.68) | 77.92 (17.42) | 0.2762 | 1 (0.51) |
| Crackles (Yes) | 140 (71.07) | 68 (69.39) | 72 (72.73) | 0.6053 | 1 (0.51) |
| Wheezing (Yes) | 32 (16.24) | 19 (19.39) | 13 (13.13) | 0.2339 | 1 (0.51) |
| Murmurs (Yes) | 60 (30.46) | 29 (29.59) | 31 (31.31) | 0.7930 | 1 (0.51) |
| Third Tone (Yes) | 3 (1.52) | 2 (2.04) | 2 (1.01) | 0.6212 | 1 (0.51) |
| *Laboratory test results* |  |  |  |  |  |
| Blood glucose* | 145.08 (52.88) | 146.59 (54.27) | 143.56 (51.68) | 0.7065 | 0 (0) |
| Urea* | 66.05 (35.43) | 69.98 (35.49) | 62.12 (35.13) | 0.0421 | 0 (0) |
| Creatinine* | 1.27 (0.57) | 1.33 (0.6) | 1.22 (0.54) | 0.0972 | 1 (0.51) |
| Glomerular filtration rate (CKD-EPI)* | 56.35 (22.50) | 53.86 (21.65) | 58.85 (23.17) | 0.1115 | 0 (0) |
| Troponin¥ | 32 [20-54] | 32 [22-50] | 31 [18.5-62] | 0.6839 | 93 (46.97) |
| Nt-proBNP¥ | 4056 [2329-8611] | 5310 [2363-10473] | 3709 [2176-5831] | 0.1335 | 83 (41.92) |
| CK* | 88.90 (53.06) | 78.63 (48.66) | 100.64 (55.97) | 0.0176 | 108 (54.55) |
| Sodium* | 139.40 (4.84) | 139.13 (5.23) | 139.67 (4.43) | 0.8884 | 2 (1.01) |
| Potassium* | 4.78 (3.38) | 4.58 (0.62) | 4.98 (4.75) | 0.4934 | 9 (4.55) |
| Albumin* | 4.02 (0.32) | 4.05 (0.29) | 3.98 (0.34) | 0.5533 | 129 (65.15) |
| CRP¥ | 5.8 [1.5-19.39] | 5.20 [1.45, 19.10] | 6.10 [1.60, 19.39] | 0.8546 | 23 (11.62) |
| Leukocytes* | 8.83 (3.50) | 9.35 (4.23) | 8.29 (2.439 | 0.1968 | 6 (3.03) |
| Haemoglobin* | 13.04 (9.06) | 13.45 (12.58) | 12.60 (1.75) | 0.1454 | 4 (2.02) |
| Hematocrit* | 39.01 (5.42) | 38.48 (5.28) | 39.59 (5.52) | 0.1750 | 4 (2.02) |
| Lymphocytes* | 1.57 (1.28) | 1.66 (1.65) | 1.48 (0.77) | 0.4152 | 14 (7.07) |
| Fibrinogen* | 401.62 (118.82) | 391.96 (127.54) | 415.84 (106.39) | 0.5757 | 151 (76.26) |
| pH* | 7.42 (0.07) | 7.43 (0.08) | 7.42 (0.06) | 0.0314 | 58 (29.29) |
| PCO2* | 41.68 (9.50) | 40.97 (10.17) | 42.43 (8.75) | 0.2625 | 58 (29.29) |
| PO2* | 70.33 (29.53) | 73.59 (30.87) | 66.87 (27.84) | 0.0658 | 58 (29.29) |
| O2 saturation* | 91.10 (9.28) | 91.13 (11.43) | 91.06 (6.27) | 0.1018 | 59 (29.80) |
| FiO2* | 23.98 (15.33) | 25.92 (20.19) | 21.41 (1.92) | 0.7415 | 147 (74.24) |
| Bicarbonate* | 26.84 (4.54) | 26.73 (4.39) | 26.96 (4.72) | 0.9486 | 61 (30.81) |
| *Etiology of HF* |  |  |  |  |  |
| Myocardial disease (Yes) | 93 (46.97) | 48 (48.48) | 45 (45.45) | 0.6692 | 0 (0) |
| Coronary artery disease (Yes) | 34 (17.26) | 15 (15.31) | 19 (19.19) | 0.4705 | 1 (0.51) |
| Hypertensive heart disease (Yes) | 47 (23.86) | 26 (26.53) | 21 (21.21) | 0.3812 | 1 (0.51) |
| Cardiomyopathy (Yes) | 46 (23.35) | 25 (25.51) | 21 (21.21) | 0.4759 | 1 (0.51) |
| Valvular disease (Yes) | 79 (39.9) | 42 (42.42) | 37 (37.37) | 0.4681 | 0 (0) |
| Arrhythmias (Yes) | 60 (30.46) | 29 (29.59) | 31 (31.31) | 0.7930 | 1 (0.51) |
| Conduction Disorders (Yes) | 4 (2.02) | 1 (1.01) | 3 (3.03) | 0.6212 | 0 (0) |
| Causes of high cardiac output (Yes) | 2 (1.01) | 2 82.02) | 0 (0) | 0.4975 | 0 (0) |
| Pericardial diseases (Yes) | 0 (0) | 0 (0) | 0 (0) | Na | 1 (0.51) |
| Diseases of the endocardium (Yes) | 0 (0) | 0 (0) | 0 (0) | Na | 0 (0) |
| Congenital heart disease (Yes) | 0 (0) | 0 (0) | 0 (0) | Na | 1 (0.51) |
| Volume Overload (Yes) | 0 (0) | 0 (0) | 0 (0) | Na | 0 (0) |
| Other (Yes) | 11 (5.56) | 5 (5.05) | 6 (6.06) | 0.7564 | 0 (0) |

*¥Results shown as mean (standard deviation). ¥Results shown as median [interquartile range]. Na: It is not possible to calculate the p-value. HF: heart failure. EF: ejection fraction.
